# Use of a Gemini-Surfactant Synthesized from the Mango Seed Oil as a CO_2_-Corrosion Inhibitor for X-120 Steel

**DOI:** 10.3390/ma14154206

**Published:** 2021-07-28

**Authors:** E. Sanchez-Salazar, E. Vazquez-Velez, J. Uruchurtu, J. Porcayo-Calderon, M. Casales, I. Rosales-Cadena, R. Lopes-Cecenes, J. G. Gonzalez-Rodriguez

**Affiliations:** 1 Engineering and Applied Sciences Research Center, Autonomus University of Morelos Sate, AV. Universidad 1001, Cuernavaca 62209, Mexico; ema.san.sal@hotmail.com (E.S.-S.); juch25@uaem.mx (J.U.); jporcayoc@gmail.com (J.P.-C.); faye12@uaem.mx (I.R.-C.); 2 Institute of Physical Science, National University Autonomous of Mexico, Mazatlán 82000, Mexico; velez.edna@icf.unam.mx (E.V.-V.); mcasales@icf.unam.mx (M.C.); 3 Metalurgy and Chemical Engineering Department, Sonora University, Hermosillo, Sonora 83000, Mexico; 4 Chemical Science and Engineering Faculty, Autonomous University of Morelos Sate, AV. Universidad 1001, Cuernavaca 62209, Mexico; rlopez@uaem.mx

**Keywords:** mango seed, CO_2_ corrosion, gemini surfactant

## Abstract

A gemini surfactant imidazoline type, namely N-(3-(2-fatty-4,5-dihydro-1H-imidazol-1-yl) propyl) fatty amide, has been obtained from the fatty acids contained in the mango seed and used as a CO_2_ corrosion inhibitor for API X-120 pipeline steel. Employed techniques involved potentiodynamic polarization curves, linear polarization resistance, and electrochemical impedance spectroscopy. These tests were supported by detailed scanning electronic microscopy (SEM) and Raman spectroscopy studies. It was found that obtained gemini surfactant greatly decreases the steel corrosion rate by retarding both anodic and cathodic electrochemical reactions, with an efficiency that increases with an increase in its concentration. Gemini surfactant inhibits the corrosion of steel by the adsorption mechanism, and it is adsorbed on to the steel surface according to a Langmuir model in a chemical type of adsorption. SEM and Raman results shown the presence of the inhibitor on the steel surface.

## 1. Introduction

The problems caused for carbon steel in the oil and gas industry due to carbon dioxide (CO_2_) corrosion is still a serious and costly problem [1]. Despite its high susceptibility to this kind of problem, such a steel is extensively used in this industry [2]. The reason is that this steel has a low cost and high level of availability. CO_2_, which normally is present in the fluid produced in the form of dissolved gas, is an influential component in oil field production fluids and it allows the formation of carbonic acid, a weak acid which can result in significant levels of corrosion, unless appropriately mitigated [3,4,5]. One of the most commonly used ways to combat this problem is the application of inhibitors [6,7] and the most commonly used include predominantly of nitrogen-containing compounds (imidazolines, amines, amides, and quaternary ammonium salts) which function by stablishing a protective film on the metal surface [8,9,10]. Surfactants, or surface active agents, are a general class of organic chemical compounds of amphiphilic molecules, each of them contains a hydrophobic (non-polar) tail and a hydrophilic (polar) head [11]. Usually, the hydrophilic head (either polar or ionic group) of surfactant molecule attaches to the metal surface and its tail or hydrophobic moiety extends away from the interface towards the solution bulk forming an array of hydrophobic tails which leads to a change in the electrochemical behavior of metal (increasing the corrosion resistance) [12]. Gemini surfactants are a class of surfactants in which the molecule is composed of two identical molecules of ordinary surfactant linked together via a linkage called spacer [13]. This spacer may be hydrophilic or hydrophobic, short or long, and rigid or flexible [14]. Gemini surfactants have been used as corrosion inhibitors for steel in different environments [15,16,17,18,19,20,21,22]. For instance, in [16] three imidazoline gemini surfactants were used as corrosion inhibitors for X-70 carbon steel in NaCl solution. Similarly, in [17] [C14-4-C14im]Br_2_ imidazolium gemini surfactant was used to inhibit the corrosion for A3 carbon steel in HCl solutions. Alternatively, propanediyl-1,3-bis(N,Ndimethyl-N-dodecylammonium bromide) and propanediyl-1,3-bis(N,N-dihydroxyethyl-N-dodecylammonium bromide) were used by Yin et al. as corrosion inhibitors for carbon steel in 1 mol HCl solution [18]. In a similar way, Abdallah et al. [19] evaluated a cationic gemini surfactant as a safe corrosion inhibitor for carbon steel in hydrochloric acid solution. Finally, Deyab and Mohsen evaluated cationic gemini surfactant, 1,2-bis(dodecyldimethylammonio) ethane dibromide (DMAEB), as corrosion inhibitor for N80 C-steel pipe in acid washing solution (15.0% HCl). Usually, electrochemical techniques such as potentiodynamic polarization curves and electrochemical impedance spectroscopy in combination with gravimetric weight loss techniques were used, obtaining inhibitor efficiency values higher than 90%.

Different research works have been performed in order to evaluate this type of compounds in combating carbon steel corrosion in a CO_2_-containing environment [23,24,25,26]. However, one restriction of these organic compounds is that many of these derivatives do not have a particularly favorable environmental profile, since they are toxic imposing, thus, restrictions on their use [27]. During last 10 years, a lot of research work has been undertaken in many naturally occurring environmentally friendly which are extracts of plants fruits, leaves, roots, flowers, etc. which have been called green inhibitors [28,29,30,31,32,33]. As a result, green chemistry and the development of efficient, environmentally friendly corrosion inhibitors has become a significant point of focus within the oil and gas industry [34].

Different organic compounds such as amides, amines or imidazolines have been extracted from different agro-industrial wastes such as coffee bagasse, coconut, avocado and rice brain and used as corrosion inhibitors for steel in acidic environments including CO_2_ [35,36,37,38]. Mexico is the fifth-largest mango producer in the world, with a volume of 1.88 million tons with an annual growth rate of 3.8%. The Guerrero region is the main producer of mango in Mexico, with 22% of the total volume production at a national level. Mango fruit (*Mangifera Indica* L.) is one of the most extensively exploited fruits for food, juice, flavor, fragrance, and color. The Mango seed is usually disposed after consumption or industrial processing. Due to the large utilization of mango fruits, more than one million tons of mango seeds are being produced as waste annually [39]. These seeds could be utilized to extract oil and birth the production of new products.

Mango seed is a good source of high-quality fat, and studies have indicated a wide variation in the fatty acid composition of oil depending on the stage of maturity [40,41]. The use of oils containing fatty acids such as oleic, linoleic, palmitic, stearic has been used to synthesize organic inhibitors such as imidazolines or amides [42]. However, it has been reported that the efficiency of inhibition may be increased by increasing the number of substituents of the functional groups and the nature of the electron donor [43] and the presence of double bonds in the hydrocarbon chain [44]. Recently, we reported the research of the use of a gemini surfactant obtained from wasted avocado oil as a green inhibitor for the CO_2_ corrosion of X-52 steel [36]. Thus, the goal of this research work is to evaluate the use of a gemini surfactant imidazoline kind obtained from mango seed as a green inhibitor for the CO_2_ corrosion of X-120 steel. In addition to this, and by comparing with previously published results [35,36,37,38], to try to find a correlation between the alkyl chain length, the presence of double bond of the hydrophobic chain of imidazoline and its inhibition performance. 

## 2. Experimental Procedure

### 2.1. Testing Material

For the present research, an API X-120 high strength pipeline steel with a chemical composition as given in Table 1 was used. Specimens measuring 10 × 10 × 5 mm were cut and encapsulated in commercial polymeric resin, abraded with 600 grade emery paper, washed and degreased with acetone.

### 2.2. Oil Extraction from MangoSseed

Five kinds of mango were collected from the Guerrero region in Mexico. The seeds were separated, washed with tap water, and dried in a hot oven over 4 days, at 60 °C, until constant weight. Seeds were ground using a blending machine. The mango seed powder (30 g) was placed on a Soxhlet system with n-hexane in a mass relationship of 1:7 respectively and the solvent was refluxed during six hours. The solvent was distilled in a rotavapor, and the quantity of oil was measured by weighing. The oil extraction optimization process was performed for the parameters of time, solvent volume, and particle size.

### 2.3. Characterization of Mango Seed Oil

Mango seed oil was characterized by Infrared Spectroscopy in an IFS 125HR FTIR Bruker Spectrometer (Billerica, MA, USA). To know the fatty acid contained in the oil, fatty acid methyl esters (FAME) were prepared by the transesterification of triglyceride. In a flask 4 g of mango oil was placed and a KOH-methanol (0.06 g in 2 mL) solution was added drop by drop at 70 °C stirring during 30 min. After this time, the glycerol was separated by density difference. The fatty acid composition was determined by Mass Gas Chromatography in an Agilent Technology 6890 Gas Chromatograph coupled to a 5973N mass detector, with an ionization mode by Electronic Impact (IE) (Santa Clara, CA, USA). The oven temperature was at 180 °C by 15 min, followed by a temperature gradient at 8 °C/min up to 250 °C/5 min.

### 2.4. Synthesis of N-(3-(2-fatty-4, 5-dihydro-1H-imidazol-1-yl) propyl) Fatty Amide

**Figure 1:**
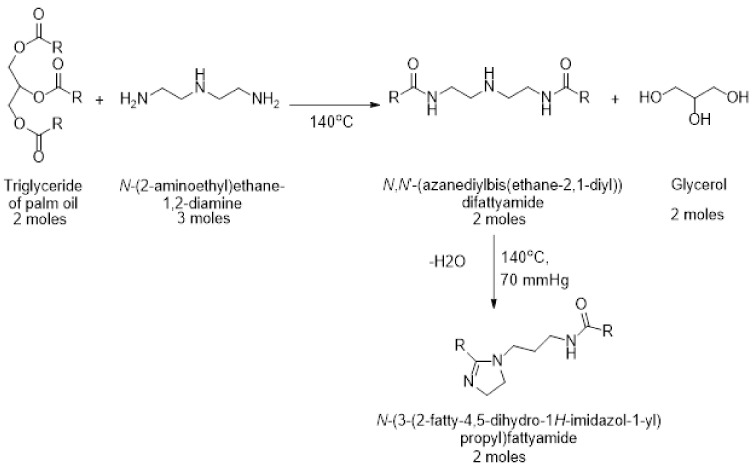
Synthesis of gemini surfactant imidazoline type. R= alkyl chains of fatty acids of mango seed oil.

The gemini surfactant corrosion inhibitor was prepared in two-steps. First, the aminolysis direct of mango seed oil with N, N-Diethylentriamine; we reported this synthetic process previously [36]. Second, the cyclization for the formation of the imidazoline ring under reduced pressure. Thin-layer chromatography indicated almost the ring cyclization complete. The compound was not purified; this was characterized by infrared spectroscopy in an IFS 125HR FTIR Brucker Spectrometer (Billerica, MA, USA) and by H1 NMR in a Varian Mercury Plus 400MHz-3mm (Palo Alto, CA, USA). The synthesis schema is shown in Figure 1. Obtained inhibitor was added to a 3.5 (wt %) NaCl solution continuously bubbled with CO_2_ gas during the whole testing time. Specimens were immersed in the solution 2 h after the bubbling was started. Testing inhibitor concentrations were, in ppm, 0, 5, 10, 25, 50, and 100 ppm, which are within the recommended commercial imidazolines but much lower to those used in some other research works using gemini surfactant type imidazoline [16] where concentrations between 100–500 ppm were used.

### 2.5. Electrochemical Tests

As reported in many research works dealing with CO_2-_containing environment [23,24,25,26], electrochemical techniques were used whereas gravimetric tests were not performed. For electrochemical testing, a Potentiostat/Galvanostat from ACM Instruments (Grange-over-sands, UK) was used. A conventional three electrodes glass cell was used, using a saturated calomel electrode (SCE) and a graphite rod as reference and auxiliary electrodes respectively. Before starting the experiments, the open circuit potential value (OCP) was allowed to reach a more or less stable value. Employed techniques were potentiodynamic polarization curves, linear polarization resistance (LPR), and electrochemical impedance spectroscopy (EIS) measurements. All tests were conducted at 50 °C. For polarization curves, scanning was carried out at a scan rate of 1.0 mV/s starting at a potential value of −250 mV more cathodic and finished at a potential value of +250 mV with respect to the free corrosion potential value (E_corr_). Corrosion current density values, I_corr_, were calculated by using Tafel extrapolation. The LPR experiments were carried out by polarizing the specimens ± 15 mV around the E_corr_ value at a scan rate of 1.0 mV/s every 60 min over 24 h to allow the calculation of the polarization resistance value, R_p_. Finally, EIS measurements were performed by applying a signal of ±15 mV peak-to-peak around the E_corr_ value in the frequency interval from 0.01 to 20,000 Hz. Corroded specimens were analyzed in a low vacuum scanning electronic microscope (SEM) (LEO, Mexico City, Mexico) whereas chemical microanalysis of corrosion products was done by using as energy dispersive spectroscope analyzer (EDX) attached to the SEM. Raman spectra were acquired to analyze the composition of the corrosion products with a Bruker Senterra II spectrometer equipped with confocal microscopy (50±) (Billerica, MA, USA). Raman excitation was performed with a laser source of 785 nm wavelength, a laser power of 1 mW and a laser spot the diameter of 15 μm. Each data collection time was 120 s.

## 3. Results and Discussion

### 3.1. Extraction of Mango Seed Oil and Optimization Parameters

The oil extraction process of mango seeds was optimized only for the *“panameño”* mango seed, which presented the best oil yield; these results are shown in Table 2. It was observed that the seed size affected the oil yield. When the oil extraction was done at the different sizes of particles, the oil yield was the highest performance (14.95%). This result is due to the containing “*Chalazal*” part of the seed coat, which is dark brown. This part is 40% of the seed coat total [45]. This part was the biggest and little bit particle, which was separated when the powder is sieved to two sieves (1.19 and 0.45 mm). So, when the particle size is the smallest (0.45 mm) increases by 2% of yield, to the fact that smaller particles have a larger amount of surface area. The oil yield increased as the extraction time increased, but it is no significative after 7 h. 

Finally, oil yield increased as the volume of n-hexane was increased in a relationship mass: volume of 1:3, 1:5, 1:7, and 1:9. However, the increase in the volume of n-hexane was not as significant as the temperature. The highest percentage oil yield was obtained with a 1:7–1:9 ratio of mass and volume of n-hexane. The oil yield results are similar to those reported in other studies [46]. The total fatty acid composition of the oil extracts of mango seed obtained in this study are shown in Table 3. The major fatty acids found were oleic acid (49%), stearic acid (33%), palmitic acid (10%), linoleic acid (4%), arachidic acid (3%), and other acids below 1%. The content oleic acid for “*panameño*” mango oil is higher than reported in other studies, where stearic acid is the most abundant one [46,47]. The major alkyl chain, oleic acid, has 18 atoms of C and one double bond, whereas stearic acid also has 18 atoms of C but has no double bonds; on the other hand, palmitic acid has 16 atoms of C and no double bond. On the other hand, gemini surfactant obtained from wasted avocado oil [36] contained alkyl chains included oleic acid (57%), palmitic acid (16%), and linoleic acid (14%). Thus, the major difference in these two extracts is that in the mango extract there is stearic acid. In contrast, in the wasted avocado extract, there is linoleic acid in a much higher amount 14% than mango extract. Summarizing, in the mango extract there is only one alkyl chain with one double bond. In contrast, the avocado extract has two alkyl chains with one and two double bonds. In both extracts, the major components have either 16 or 18 atoms of C.

Corrosion inhibitor derived from avocado oil reported previously is also a gemini surfactant with a different molecular structure. The first step in the synthesis of both inhibitors is through an aminolysis process. The inhibitor derived from mango seed oil involves a second reaction step: the cyclization to imidazoline. This reaction is not completed to 100%. However, unlike the amide group, imidazoline presents unshared pairs of electrons from heteroatom and p orbitals from the heterocycle, which has a better interaction with the d orbitals of metallic surface. However, the originality of this article is not based only on the imidazoline molecule but in mango seed oil as a source of raw material. Nowadays, sustainable and environmentally friendly sources must be searched, which replace inhibitors derived from petroleum. Mango seed is an industrial waste that can present added value by extracting its oil and forming other compounds such as biodiesel or formulations for cosmetic use.

Generally, this type of inhibitors, due to their stability, are the most reported in their evaluation at high temperatures in sweet corrosion [48]. The inhibition efficiency increased with the length of the alkyl chain to foster the formation of larger and more stable lamellar micelles that exhibited stronger interactions with the steel surface. Nevertheless, gemini surfactant has indicated better film persistency than conventional surfactant by forming a bi-/multilayer film [49]. However, imidazoline derivatives surfactants have exhibited poor chemical stability, given the tendency to hydrolyze into their amide precursors under sweet corrosion conditions. However, this phenomenon has caused fatty amides to show higher inhibition efficiency [50]. Thermal stability studies (DSC-TGA) of fatty amides derived from vegetable oils show that their weight remains constant at temperatures below 200 °C and the evaporation temperature increases as the alkyl chain [51,52].

### 3.2. Characterization of Mango Oil and Corrosion Inhibitor

**Figure 2:**
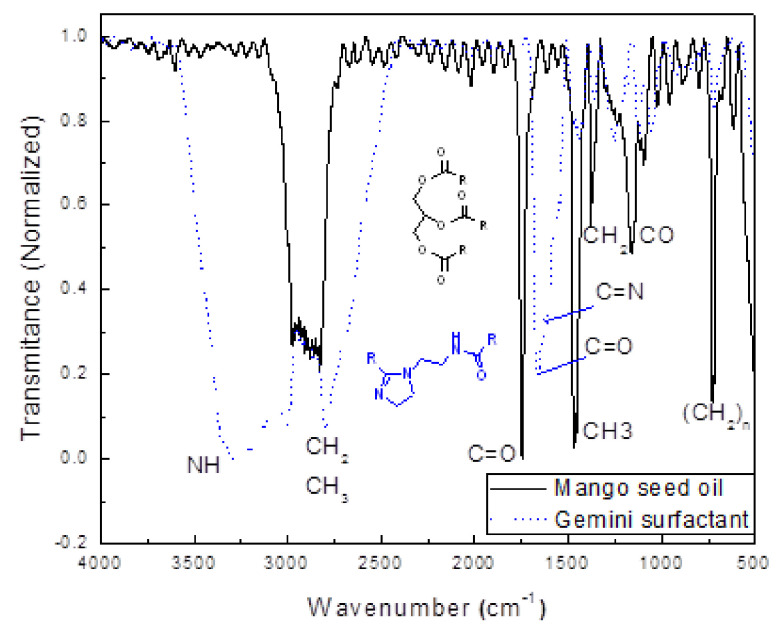
Infrared spectra of mango seed oil and Gemini surfactant.

The use of IR spectroscopy is ideal for triglycerides characterization and their derivate products as a mixture. Figure 2 shows the infrared spectra of triglycerides contained in the mango oil and the gemini surfactant.

**Figure 3:**
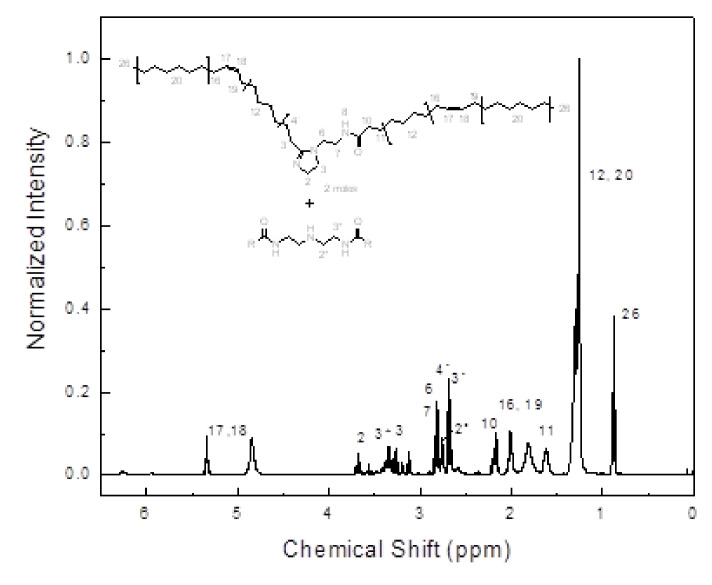
H NMR spectrum of gemini surfactant.

For oil, the spectrum presents a sharp and strong band corresponds to the C=O stretch vibration of the ester group at 1746 cm^−1^, which is displaced to 1658 cm^−1^ for the amide group of the gemini surfactant. The C-H stretch vibration of methyl and methylene groups of the alkyl chains appears at 2894 cm^−1^ and the deformation vibration at 1466, 1378, and 718 cm^−1^ for the CH_3_, CH_2_, and (CH_2_)n groups respectively. A band for the C-O stretch of the glycerol group in the triglyceride is observed at 1162 cm^−1^ also. In a region between 1252 and 1111 cm^−1^ the C-N stretch vibration corresponding to the amide and imidazoline groups in the gemini surfactant appeared. The band corresponding to the N-H stretch vibration of secondary amide appears at 3293 cm^−1^. Finally, for the C=N stretch vibration of the imidazoline ring appears at 1619 cm^−1^. The spectrum of gemini surfactant presents the band characteristic of the O-H group in the region between 300–3300 cm^−1^ due to the presence of the sub-product glycerol. This spectrum is similar to reported for a corrosion inhibitor imidazoline type [53]. Gemini surfactant was characterized by H1 spectroscopy, as given in Figure 3, where it can be seen that the main compound is the imidazoline derived from oleic acid. It is observed a relationship 2:1 between the imidazoline and its diamide precursor. This relationship was measured by the integration of signal in 2.18 ppm, which corresponds to the α-CH_2_ (2*) to the amine group, this integration is for two protons instead of four, compared with the signal α-CH_2_ (6) that is integrated by two protons of the imidazoline ring. The glycerol subgroup at 3.4 and 3.57 ppm (CH_2_, CH) can be observed also in a much lower amount. The chemical shift 1H RMN (400 MHz, CDCl3 δ = 7.3): δ = 0.76 (t, J = 6.8 Hz, 6 H (26)), 1.22 (m, 58 H (12,20)), 1.61 (t, J = 7.6 Hz, 4 H (11), 1.81 (s, 8H (16, 19)), 2.17 (m, 4H (10)), 2.68 (m, 4 H (4’,3’)), 2.82 (m, 4 H (6,7)), 3.12 and 3.18 (t, J = 6.24 Hz, 2H (3)), 3.36 (3*), 3.68 (t, J = 9.36 Hz, 2H (2)), 4.85 (N-H), 5.34 (td, 4H (17, 18)).

### 3.3. Potentiodynamic Polarization Curves

**Figure 4:**
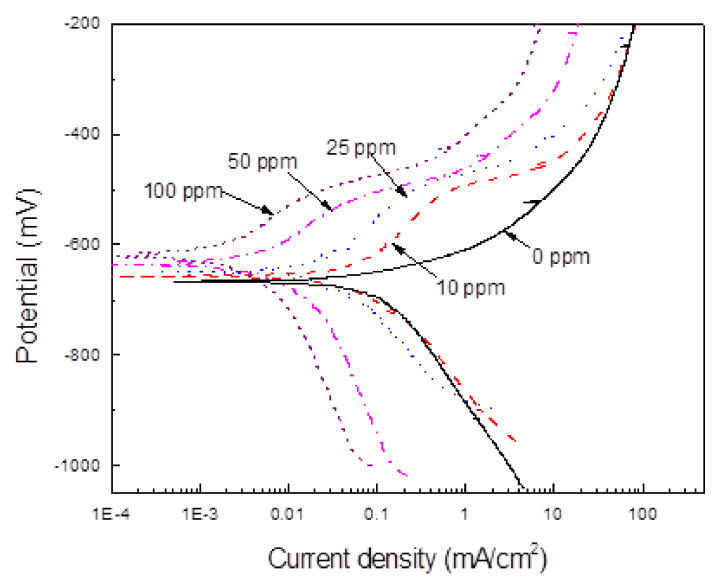
Effect of gemini surfactant concentration on the polarization curves for X-120 steel in a CO_2_-saturated NaCl solution at 50 °C.

Polarization curves for X-120 steel exposed to a CO_2_-saturated NaCl solution containing different concentration of the gemini surfactant are shown in Figure 4 and their electrochemical parameters are given in Table 4. In absence of inhibitor, curve displays an active behavior only, without evidence of the formation of a passive layer. However, with the addition of the inhibitor, polarization curves display an active-passive behavior. It can be seen that the addition of the inhibitor shifted the E_corr_ value towards nobler values, and the higher the inhibitor concentration, the nobler the E_corr_ value become; however, the difference between the most active and the noblest E_corr_ values was 60 mV only. The addition of the inhibitor decreased both anodic and cathodic current density values and in the corrosion current density also, since it was decreased from a value of 0.12 mA/cm^2^.

In absence of inhibitor, to a value of 0.001 mA/cm^2^ when 100 ppm of Gemini surfactant were added. Inhibitor efficiency, I.E., which were calculated according to

(1) $$I.E\;\left(\%\right)=\left(\frac{I_{\mathrm{corr}}-I_{\mathrm{corr}}^{\prime }}{I_{\mathrm{corr}}}\right)\times 100$$

where I_corr_ and I′_corr_ are the corrosion current density values in absence and presence of the gemini surfactant respectively. From data given in Table 4, it can be observed an increase in the inhibitor efficiency value with an increase in its concentration. For the gemini surfactant obtained from wasted avocado oil [36], with two alkyl chains with one double bond, the lowest obtained I_corr_ value was 0.006 mA/cm^2^, higher than the one obtained in the present work, also with the addition of 25 ppm of inhibitor. Thus, it seems that the presence of more alkyl chains on the extract with double bonds is detrimental on the inhibitor performance. On the other hand, the alkyl chains length was very similar to each other, between 16 and 18 atoms of C, and there is no clear effect on this length and the metal corrosion rate. Since the metal area covered by the inhibitor, θ, which was calculated by dividing the inhibitor efficiency by 100, increases in the same fashion as the inhibitor efficiency value, it is concluded that the decrease in the I_corr_ value is due to the adsorption of the inhibitor on to the steel area surface and to an increase in the inhibitor concentration brings an increase of the steel area covered by the inhibitor. Electrochemical reactions that take place in the corrosion of steel in CO_2_ includes: (a) as anodic reaction, the dissolution of iron; and (b) as cathodic reactions there are two: hydrogen evolution as well as the dissociation of carbonic acid (H_2_CO_3_) [54,55] in to protons, carbonate, and bicarbonate ions [56,57]. From Table 4, it can be seen that the addition of the gemini surfactant affects both anodic and cathodic Tafel slopes, however, the cathodic Tafel slope was much more affected than the anodic one, therefore it can be said that the gemini surfactant acts as a mixed type of inhibitor, with a more pronounced effect on the cathodic reactions, by blocking effectively the hydrogen evolution.

**Figure 5:**
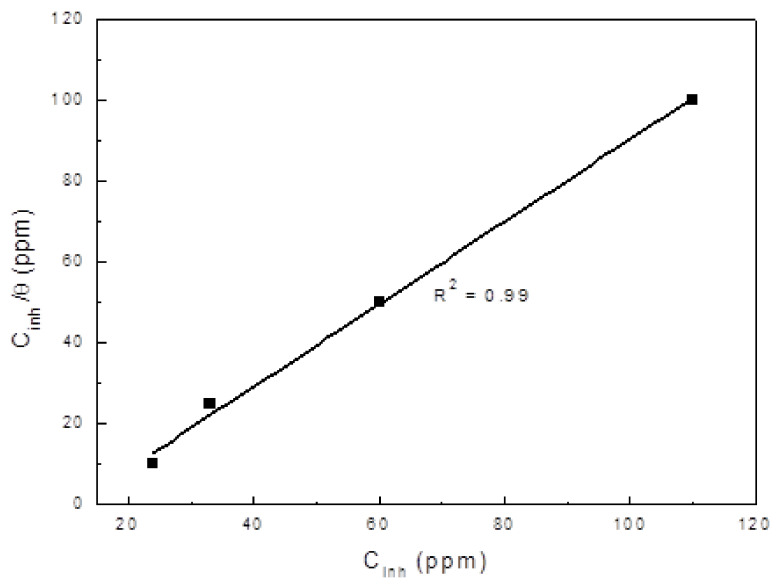
Langmuir adsorption isotherm for X-120 steel in a CO_2_-saturated NaCl solution containing different concentrations of gemini surfactant.

The reason why the corrosion rate of X-120 steel decreases in the CO_2_-containing NaCl solution with the addition of the inhibitor, as we have explained above, is because the inhibitor is adsorbed on to the steel surface to form a protective, passive layer of corrosion products, as shown by polarization curves in Figure 4. The way it adorbs on to the steel surface can be clarified by the use of the adsorption isotherms. Different adsorption models exist, including the Langmuir, Tempkin, Frumkin, and Flory–Huggins, and, among the different models, the adsorption isotherm which had the best fitting, with a correlation factor, R^2^ = 0.99, was the Langmuir one, Figure 5. The assumptions made in the Langmuir adsorption isotherm are a) adsorption of the inhibitor is the single-layer. It means that each molecule of inhibitor is absorbed in one place and they do not fit together, b) adsorbed molecules do not have any kind of the interaction with each other; c) the surface is uniform and homogeneous. According to this isotherm, covered surface (θ) by inhibitor and the inhibitor concentration (C_inh_) are linked together by the equation carbonic acid dissociation reactions.

C_inh_/θ = 1/K_ads_ + C_inh_ (2)

where is the adsorption isotherm and is related to the standard free energy of adsorption ($\Delta G_{\mathrm{ads}}^{0}$) according to the equation

(3) $$\Delta G_{\mathrm{ads}}^{0}=-\mathrm{RT}\;\mathrm{In}\left(10^{6}\;K_{\mathrm{ads}}\right)$$

where R is the universal gas constant and T is the absolute temperature. By using data given in Figure 5, an adsorption free-energy value of −38.4 kJ mol^−1^ was obtained.

In general, two kinds of the adsorption are done by the organic molecules including the physical and the chemical adsorption. Since it is defined that the boundary between them is $\Delta G_{\mathrm{ads}}^{0}$ = −38.4 kJ mol^−1^; thus, it is found that inhibition mechanism of the extraction of mango seed is mainly chemical adsorption on the metal surface [58]. The gemini surfactant obtained from wasted avocado oil [36] was physically adsorbed to the steel surface according to a Langmuir adsorption isotherm also.

### 3.4. LPR Measurements

**Figure 6:**
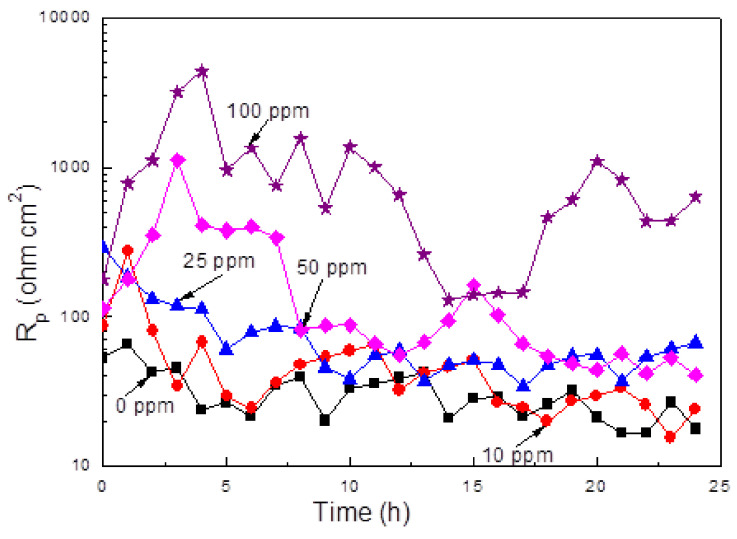
Variation on the R_p_ value for X-120 steel in a CO_2_-saturated NaCl solution containing different concentrations of gemini surfactant.

The effect of inhibitor concentration on the linear polarization resistance value, R_p_, for X-120 steel in the CO_2_-saturated NaCl solution is shown in Figure 6.

It can be seen that the R_p_ value increases with an increase in the inhibitor concentration; however, for inhibitor concentrations lower than 25 ppm, the R_p_ value decreases as time elapses which is due to several reasons. For uninhibited solution, the formed corrosion products layer of iron carbonate is not protective, and the environment continues corroding the underlying metal. On the other side, due to the fact that according to results given above, the inhibitor is adsorbed on to the steel surface in a physical, weak way, which means that the adhesion of the film to the surface metal is so weak that it cannot remain on the steel surface during a long period of time, and after a short period of time, this film is detached from the steel surface, leading to a decrease in the R_p_ value. Similar to this, as the inhibitor concentration increases, more inhibitor molecules cover the steel surface with an increase in the steel surface area covered by it, but after some time, this film remains on the steel surface for a longer period of time than that for lower inhibitor concentrations. This way, as the inhibitor concentration increase for inhibitor concentrations of 50 and 100 ppm, the time that the film formed by the inhibitor is longer. However, after a period of time, which increases with the inhibitor concentration, the R_p_ values decreases due to the corrosion products film detachment from the metal. Highest obtained R_p_ value was 4500 ohm cm^2^ after 4 h of immersion with the addition of 100 ppm, whereas for the R_p_ gemini surfactant obtained from avocado oil [36] the highest R_p_ value was 3200 ohm cm^2^. On other hand, an amide-base inhibitor obtained from palm oil contained the same alkyl chains as the one obtained from avocado, oleic, linoleic, and palmitic acid plus the saturated stearic acid—i.e., only one containing one double bond [59]—a highest R_p_ value of 9500 ohm cm^2^ was obtained. Thus, the presence of two alkyl chains with one double bond seems to be detrimental on the inhibitor performance. Chemisorption pattern of the inhibitor derived from mango seed oil in the material shows the synergy of its content of unsaturated and saturated fatty acids. The content of oleic acid (49%), stearic, and palmitic acid (33% + 10%) give it the efficacy of corrosion inhibition because molecules with oleic content are adsorbed on the surface of the material by the interaction of hydrophilic groups and the unsaturation of the alkyl chains. In contrast, the chains of saturated fatty acids form a protective double film barrier so that the water molecules do not penetrate on the surface. In the inhibitor derived from avocado oil, its content of oleic and linoleic acids (57% + 14%) is much higher than saturated fatty acids (16%). Therefore, its effectiveness at low concentrations is because the unsaturation in the alkyl chains is mainly adsorbed on the material surface.

### 3.5. EIS Measurements

**Figure 7:**
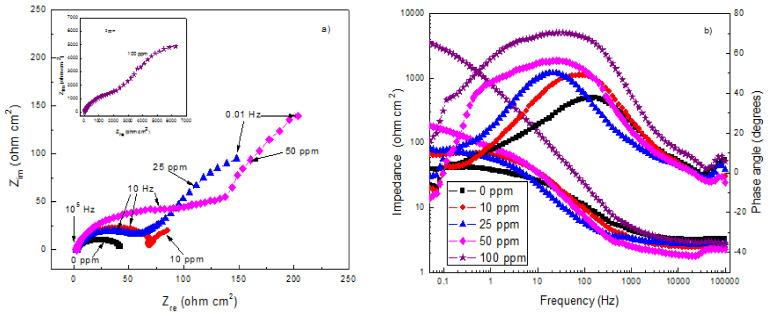
(**a**) Nyquist and (**b**) Bode diagrams for X-120 steel in a CO_2_-saturated NaCl solution at 50 °C containing different concentrations of gemini surfactant.

**Figure 8:**
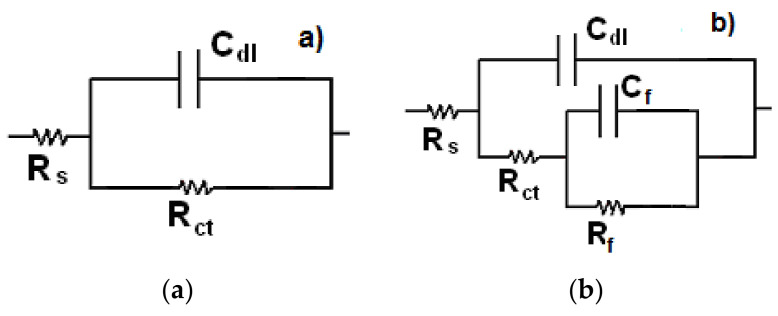
Electric circuits to simulate EIS data X-52 steel corroded in a CO_2_-saturated 3% NaCl solution (**a**) in absence and (**b**) presence of gemini surfactant.

EIS data in both Nyquist and Bode formats for X-120 steel in X-120 steel in the CO_2_-saturated NaCl solution is shown in Figure 7. For the uninhibited solution, Nyquist data Figure 7a, display a single depressed, capacitive semicircle at all the frequency values, indicating a corrosion process controlled by the charge transfer reaction. On the other hand, for inhibited solutions, Nyquist diagrams display two depressed, capacitive-like semicircles, one located at high and intermediate frequency values followed by another one at lower frequency values. The high and intermediate frequency values semicircle is related with the electrochemical reactions taking place at the metal/electrolyte interface through the double electrochemical layer, whereas the lower frequency one is related to the electrochemical reactions taking place at the metal/corrosion products interface. Generally speaking, the semicircles diameters increase with an increase in the gemini surfactant concentration. Bode diagrams, Figure 7b, show that the module value increases as the inhibitor concentration increases in the same fashion as the semicircles diameters in the Nyquist plots behave, reaching its highest value with an inhibitor concentration of 100 ppm, for more than two orders of magnitude than that obtained for the uninhibited solution; it was observed the existence of two different slopes, one at intermediate frequency values and a second one at lower frequency values, indicating, thus, the existence of two time constants. On the other hand, the phase angle increases as the inhibitor concentration increases, reaching its highest value of 60 degrees at 100 ppm. This is attributed to the decrease in surface irregularities as a result of the adsorption of organic molecules of the extracts on metal and, thus, a lower metal dissolution rate [60,61,62,63]. It is clear that in the uninhibited solution there is only one peak, and, thus, only one time constant, indicating the absence of any protective layer, whereas in presence of inhibitor, the presence of two time constants, and thus, the presence of a protective corrosion products layer, is clear. EIS data were simulated by using electric circuits given in Figure 8. In this figure, R_s_ represents the electrolyte or solution resistance, R_ct_ the charge transfer resistance, C_dl_ is the double electrochemical layer capacitance, and R_f_ and C_f_ the corrosion products film resistance and capacitance respectively.

Parameters used to fit the EIS data by using electric circuits shown in Figure 8 are given in Table 5.

Same electric circuits were used for the gemini surfactant obtained from avocado oil [36] and from palm oil [59] suggesting that the presence of two alkyl chains with double bonds did not affect the corrosion mechanism. Important to note that the corrosion products film resistance, R_f_, are bigger than those for the charge transfer resistance, R_ct_, indicating that the corrosion resistance of X-120 steel is given mainly by the corrosion products film formed by the inhibitor and iron ions released during the steel dissolution reaction. Secondly, the charge transfer resistance, R_ct_, increases with an increase in the inhibitor concentration. Simultaneously, an increase in the inhibitor concentration brings a decrease in the double electrochemical capacitance value, C_dl_. The increment in the R_ct_ value and decrement in the C_dl_ value is attributed to the formation of a protective film at the metal interface. Double-layer capacitance can be obtained from the equation

C_dl_ = εε_0_A/d (4)

where ε_o_ is the dielectric constant in the vacuum, A is the electrode area at exposing electrolyte and d is the thickness of the protective layer. Therefore, it is reasonable that the decrease in the capacitance can be occurred because of the absorption of the inhibitor and create the protective layer and/or desorption of the molecules of water with the dielectric constant more than the surface and the absorption of the inhibitor with the dielectric constant less than on the surface of the metal. The changes in both R_ct_ and C_dl_ values were caused by the replacement of water molecules by adsorption of inhibitor on the steel surface, reducing the extent of metal dissolution.

### 3.6. Surface Analysis

**Figure 9:**
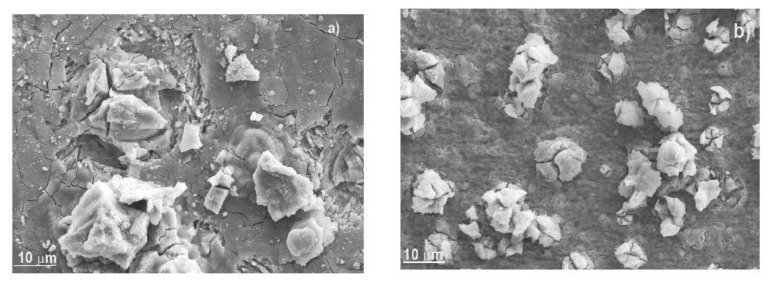
SEM micrographs of X-120 steel corroded in a CO_2_-saturated NaCl solution at 50 °C containing (**a**) 0 and (**b**) 100 ppm of gemini surfactant.

**Figure 10:**
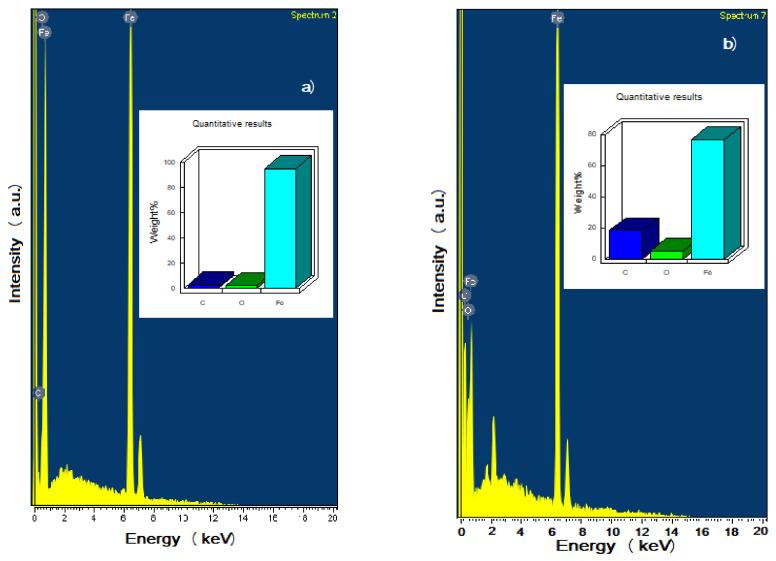
EDX micro chemical analysis of corrosion products formed on top of steel corroding in solution containing (**a**) 0 and (**b**) 100 ppm of gemini surfactant.

Micrographs of corroded specimens in the CO_2_-containing NaCl solution without and with gemini surfactant are shown in Figure 9. The film formed on steel corroded in the uninhibited solution, Figure 9a, contains many defects such as micro cracks which form path for the electrolyte get in contact with underlying metal. On the other hand, film formed on top of steel corroded in the solution containing 100 ppm of the Gemini surfactant, Figure 9b, is much more compact, and the presence of defects such as micro cracks was not evident. Thus, the paths for the entrance of the electrolyte to corrode the underlying metal were much less than that in absence of the inhibitor, indicating why the steel under these conditions exhibited a lower corrosion rate than that for steel corroded without inhibitor. On the other hand, EDX micro chemical analysis of the corrosion products show the presence of C, O, and F for steel corroded in absence of the inhibitor, Figure 10a, however, the amount of C and O increased for the specimen corroded in presence of 100 ppm of inhibitor, due mainly to the fact that these two chemical elements are present in the gemini surfactant.

**Figure 11:**
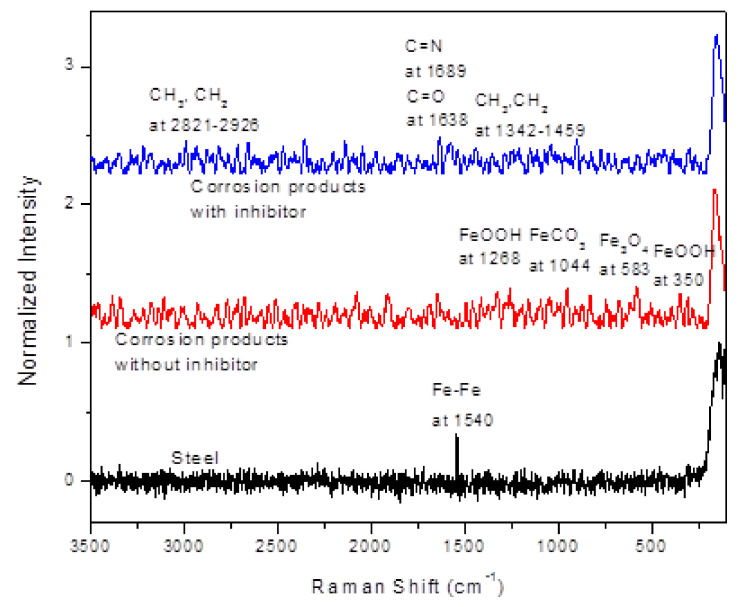
Raman spectrum of corrosion products formed on top of X-120 steel corroded in absence and presence of 100 ppm of gemini surfactant.

The use of Raman spectroscopy enabled the determination of the corrosion products film on the X-120 steel surface corroded in absence of the gemini surfactant. The spectrum in Figure 11 shows the peaks of corrosion products in absence and presence of the inhibitor. For comparison, the same analysis was done for the steel alone without being exposed to the corrosion test. For the corrosion products found in absence of the inhibitor, the main peak within the spectrum at 583 cm^−1^ confirms the presence of magnetite (Fe_3_O_4_); two peaks at 350 and 1268 cm^−1^ for α and γ-FeOOH respectively [63]. Yong Hua et al. reported the FeCO_3_ crystals formed at high temperature at 290 and 1086 cm^−1^. These bands can be contained in the spectrum of corrosion products at 298 and 1044 cm^−1^. On the other hand, the corrosion products in presence of the inhibitor shows signals which correspond to the CH_3_ and CH_2_ groups at 2821–2926 cm^−1^, another at 1689 cm^−1^ which corresponds to the C=N; other signal observed at 1638 and 1341–1459 cm^−1^ which have been assigned to the C=O and CH_3_ and CH_2_ groups, all of them present in the gemini surfactant. This clear evidence that the decrease in the corrosion rate of X-120 steel n the CO_2_-containing NaCl solution is due to the adsorption of this organic compound on to the steel surface.

### 3.7. Corrosion Inhibition Mechanism by the Gemini Surfactant Derived of Mango Oil

**Figure 12:**
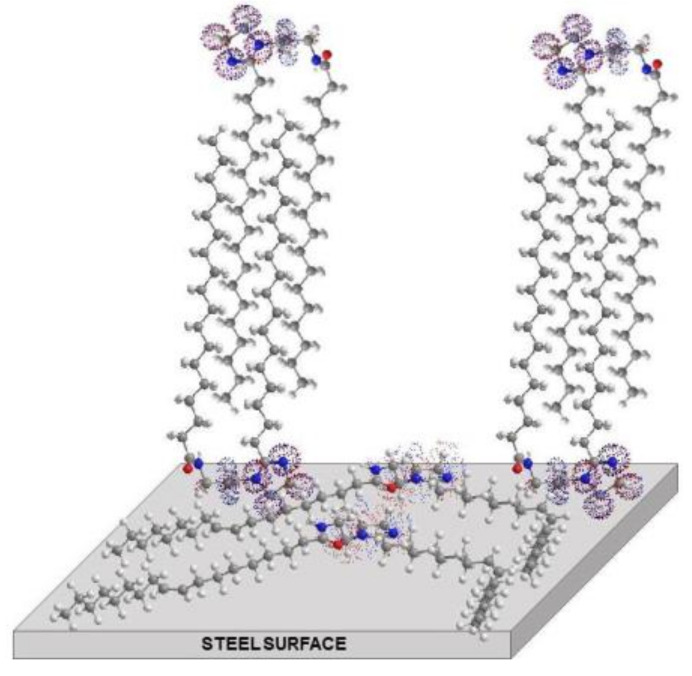
Adsorption process of the gemini surfactant on the steel surface by the synergy of their oleic, stearic, and palmitic molecules.

In order to better explain the corrosion inhibition process and mechanism of the gemini surfactant on the surface of the steel, the schematic diagram of the adsorption process is shown in Figure 12. The hydrophilic groups are adsorbed on the metal surface to occupy one adsorption site. This adsorption is preferable by an electronic interaction between the unshared pairs of electrons from the heteroatoms or the π orbitals bond from the molecule, with the d orbitals on the metallic surface. Gemini surfactants derived from mango oil mainly contain the alkyl chains of oleic (49%), stearic (33%), and palmitic (10%) acids. The adsorption of gemini oleic surfactant occurs through horizontal binding because the molecule lies on the surface due to unsaturated chains. On the other hand, the gemini stearic and palmitic surfactant occupy another adsorption site by the hydrophilic group and the vertical adsorption takes place as a result of hydrophobic interaction between saturated chains. The columnar adsorption of the surfactant continues with the hydrophilic group popeyed into the solution at higher concentration and the hydrocarbon tail mingling with the adsorbed monomers, by the hydrophobic interaction and Van der Waals forces, until the formation of a very rigid barrier of surfactant molecules on the steel surface.

## 4. Conclusions

A gemini surfactant, N-(3-(2-fatty-4, 5-dihydro-1H-imidazol-1-yl) propyl) fatty amide, has been synthetized from the fatty acids contained in the mango seed and used as CO_2_-corrosion inhibitor for API X-120 steel. Adsorption of the gemini surfactant corrosion inhibitor produced a decrease in the corrosion rate of steel and it was found to follow the Langmuir adsorption isotherm. The gemini surfactant affects the kinetics of the corrosion processes, acting as a mixed type of inhibitor, with a more pronounced effect on the cathodic reactions; its inhibition efficiencies increased with an increase in the inhibitor concentration. Thermodynamic parameters revealed that the inhibitor is chemically adsorbed on to the metal surface. Negative values for ΔG_ads_ indicates a spontaneous adsorption process of this gemini surfactant on to the steel surface. SEM and Raman spectroscopy studies showed that the corrosion inhibition by the gemini surfactant is due to its adsorption on to the steel surface to be part of the corrosion products.

## Figures and Tables

**Table 1:** Chemical composition of API X-120 pipeline steel (wt %).

**Fe**	**C**	**N**	**Si**	**Mn**	**P**	**S**	**Cr**
Bal.	0.027	0.006	0.24	1.00	0.003	0.005	0.4
**Mo**	**Ni**	**Al**	**Co**	**Cu**	**Nb**	**Ti**	
0.18	1.35	0.045	0.004	0.0107	0.024	0.154	

**Table 2:** Optimization of parameters in the oil extraction.

Mango Kind	Recovery Oil Yield (%)	Particle Size (mm)	Duration of Extraction (h)	Recovery Oil Yield (%)	M:V Relationship (g:mL)	Recovery Oil Yield (%)
Manila	10.34	2.5–0.3	6	14.95	1:7	-
Ataulfo	7.53	1.19	6	7.38	1:7	-
Haden	11.54	0.45	4	8.2	1:3	8.79
			5	8.84	1:5	8.99
Criollo	11.22		6	8.95	1:7	9.45
Panameño	14.95		7	9.45	1:9	9.49
			8	9.63	1:11	9.40

**Table 3:** Fatty acid composition of mango seed oil.

Fatty Acids	Structure	Fatty Acids (wt %)
Palmitic Acid	C16:0	10
Stearic Acid	C18:0	33
Oleic Acid	C18:1	49
Linoleic Acid	C18:2	4
Arachidic Acid	C20:0	3

**Table 4:** Electrochemical parameters obtained from polarization curves for X-120 steel immersed in a CO_2_-saturated NaCl solution containing gemini surfactant.

C_inh_ (ppm).	E_corr_ (mV)	I_corr_ (mA/cm^2^)	β_a_ (mV/dec)	−β_c_ (mV/dec)	I.E. (%)	θ
0	−670	0.12	45	230	-	-
10	−650	0.07	110	180	41	0.41
25	−635	0.03	100	160	75	0.75
50	−620	0.007	90	150	83	0.83
100	−610	0.001	90	140	98	0.98

**Table 5:** Electrochemical parameters to fit the EIS data of X-120 steel corroded in a CO_2_-saturated 3% NaCl solution containing different concentrations of gemini surfactant.

C_inh_ (ppm)	R_s_ (ohm m^2^)	C_dl_ (μF cm^−2^)	R_ct_ (ohm m^2^)	C_f_ (μF cm^−2^)	R_f_ (ohm m^2^)
0	3.2	2.3 × 10^−4^	43	-	-
10	2.8	1.4 × 10^−4^	55	6.3 × 10^−4^	175
25	2.7	7.5 × 10^−5^	69	1.2 × 10^−4^	310
50	2.4	3.1 × 10^−5^	132	7.05 × 10^−5^	450
100	3.1	5.5 × 10^−6^	2100	1.4 × 10^−5^	7100

## Data Availability

The data presented in this study are available on request from the corresponding author.
